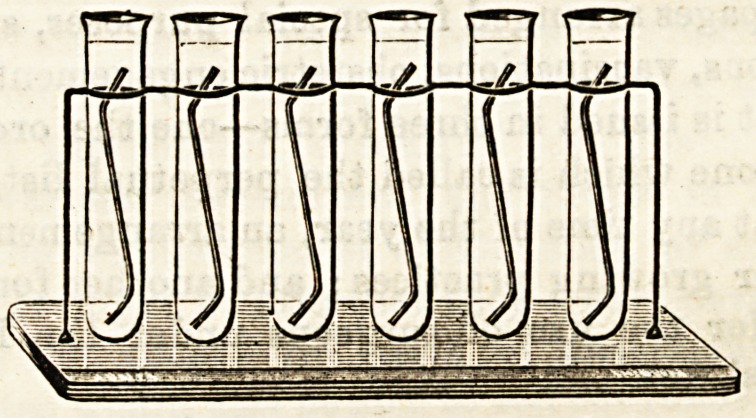# New Appliances and Things Medical

**Published:** 1898-11-19

**Authors:** 


### NEW APPLIANCES AND THINGS MEDICAL.

[We shall be glad to receive, at our Office, 28 & 29, Southampton Street, Strand, London, W.C., from the manufacturers, specimens of all new preparations and appliances which may be brought out from time to time.]

### THE "GNOM" PATENT DOMESTIC STERILISER.

(Lumley and Co., 1 America Square, Minories, London, E.C.)

One could not well have a more simple means of sterilising milk than is here provided. It consists of an enamelled iron pan, about the size of, and in fact very like, a housemaid's pail. Into this is placed a frame containing a number of bottles each holding about half a pint of milk. These bottles are closed by stoppers held in place by a very efficient spring wire arrangement. The pan is provided with a lid. When it is to be used the bottles are filled with milk, the stoppers are closed, the bottles are placed in their frame, and then are lowered all together into the pan. This is then filled up with water to within an inch of the top of the bottles, and placed on a stove or fire till the water boils. When this happens the lid is put on and the boiling is continued for ten minutes. The whole apparatus is then allowed to cool. It is stated that the milk will then be sterilised, and fit to keep any time. Whether this will be the case in every instance we are not prepared to say. Clearly, the milk will never quite reach the boiling point of water, so that we cannot look for its absolute sterilisation if it should happen to contain resistent spores. But in practice such spores need hardly be considered, the adult forms of bacteria being all that we have to deal with, and these will easily be killed off by the temperature attained. It is important, however, that the directions should be strictly followed in regard to keeping up the boiling for ten minutes, otherwise the milk will not have been raised to the proper temperature and the stoppers will not be sterilised at all. Of the advantages of using such an apparatus we need say nothing; the remarks lately made by Sir Richard T. Thorne in the Harben lectures show the great loss of life which is caused every year by the use of unsterilised milk. The great difficulty is to induce a mistress or a cook to have the milk boiled. This is not due entirely to the alteration of taste produced by boiling, but to the trouble which arises from the production of scum and to the ever-present risk of burning the milk if it is not constantly watched. To boil a big pan of milk is by no means a simple process, but with such an apparatus as this all is easy. Practically no scum is formed if the bottles are well filled, while all chance of burning is absolutely abolished, and as a reward for her trouble the cook finds that she is relieved from the nuisance of being dependent upon the milkman's call, for if she always keeps a few bottles on hand she never need be short of milk.

### ASEPTIC FEMALE CATHETER STAND.

(Reynolds and Branson, Ltd., Leeds.)

This is a stand for half a dozen large, strong glass test tubes, each of which is intended to hold one glass female catheter, buried overhead in an appropriate antiseptic solution. The rings by which the tubes are held are of metal, and the base is of thick plate-glass, a strip along the front of which is frosted, so that the name of the patient for whom each catheter is reserved may be written opposite its proper test tube. It is a good idea well carried out. We are afraid that it will be found very easy to break the catheters in popping them into their places, but if a fairly strong antiseptic is to be used there ought not to be any objection to placing a little cotton wool in the bottom of the tube each time it is refilled. Of course, this catheter stand is intended for use in a female ward, but if one were allowed to divert an invention from its proper purposes, it is clear that such a stand, furnished as it is with a base which can be written upon, would do well for keeping specimens of urine for inspection. If used for this purpose, however, lipped tubes should be provided.

## Figures and Tables

**Figure f1:**